# Supplementary material for: Consumer Preference of Traditional Korean Soy Sauce (Ganjang) and Its Relationship with Sensory Attributes and Physicochemical Properties
Source: Foods. 2023 Jun 13;12(12):2361. doi: 10.3390/foods12122361 (PMC10296797; doi:10.3390/foods12122361)
Supplement: Supplementary file 1 [file foods-12-02361-s001.zip › foods-2429522-supplementary.pdf]

Table S1. Mean value of regional *ganjang* products for physicochemical properties and *p*-value derived from analysis of variance.

	**GW**	**GG**	**CC**	**JL**	**GS**	**JJ**	***p*-value**
**Total solid**	72.15	61.43	66.03	64.93	68.81	67.74	**0.310**
**Ash**	20.81	23.57	22.21	22.14	20.00	22.61	**0.431**
**Lipid**	0.32	0.41	0.38	0.41	0.47	0.39	**0.832**
**Total nitrogen**	0.63	1.30	0.94	1.03	1.02	0.82	**0.298**
**Color-L**	67.38	66.50	70.80	72.67	66.90	69.31	**0.041**
**Color-a**	2.29	0.90	1.13	0.52	2.77	0.35	**0.243**
**Color-b**	0.03	-0.12	0.18	-0.07	1.57	-0.06	**0.141**
**pH**	5.60	5.38	5.49	5.34	5.68	5.28	**0.814**
**Acidity**	0.84	1.47	1.35	1.50	1.28	1.36	**0.594**
**Salinity**	24.21	30.95	26.10	25.23	22.98	26.69	**0.109**
**Reducing sugar**	0.70	0.63	0.70	0.90	0.63	0.76	**0.818**

Note. Gangwon; GG, Gyeonggi; CC, Chungcheong; JL, Jeolla; GS, Gyeongsang; JJ, Jeju provinces.

Table S2. Mean value of regional *ganjang* products for free amino acids and *p*-value derived from analysis of variance.

	GW	GG	CC	JL	GS	JJ	*p*-value
**Ala**	141.4	596.0	295.2	315.1	412.6	232.0	**0.403**
**Arg**	14.7	34.8	19.8	52.7	30.3	23.1	**0.440**
**Asp**	62.0	142.6	109.7	169.3	123.0	184.1	**0.449**
**Cys**	0.0	6.4	1.1	0.2	1.4	1.0	**0.446**
**GABA**	18.3	455.8	129.5	90.1	144.3	62.1	**0.340**
**Glu**	263.0	351.8	516.0	576.6	519.6	553.2	**0.630**
**Gly**	56.2	90.5	104.4	109.1	131.3	104.3	**0.373**
**His**	30.5	50.3	38.9	44.1	32.4	27.4	**0.616**
**Ile**	79.5	201.7	146.1	181.0	201.3	150.1	**0.332**
**Leu**	135.7	295.1	236.9	285.7	321.4	243.1	**0.383**
**Lys**	157.9	272.0	293.1	328.2	324.7	306.6	**0.446**
**Met**	13.8	38.3	28.3	28.5	44.5	32.2	**0.528**
**Orn**	65.5	118.6	104.3	121.5	90.1	164.6	**0.621**
**Phe**	85.2	203.9	136.1	165.4	180.1	145.7	**0.499**
**Pro**	87.1	178.3	133.1	187.2	143.3	134.8	**0.353**
**Ser**	76.8	130.8	99.2	143.2	126.4	151.8	**0.760**
**Thr**	56.8	117.8	97.1	129.2	97.6	128.4	**0.613**
**Tyr**	50.3	78.5	34.3	77.0	62.6	89.2	**0.169**
**Val**	88.4	273.0	155.5	200.8	219.1	171.0	**0.414**

Note. Ala, alanine; Arg, arginine; Asp, aspartic acid; Cys, cysteine; GABA, 4-aminobutyric acid; Glu, glutamic acid; Gly, glycine; His, histidine; Ile, isoleucine; Leu, leucine; Lys, lysine; Met, methionine; Orn, ornithine; Phe, phenylalanine; Pro, proline; Ser, serine; Thr, threonine; Tyr, tyrosine; Val, valine; Gangwon; GG, Gyeonggi; CC, Chungcheong; JL, Jeolla; GS, Gyeongsang; JJ, Jeju provinces.

Table S3. Mean value of regional *ganjang* products for organic acids and *p*-value derived from analysis of variance.

	GW	GG	CC	JL	GS	JJ	*p*-value
**Citrate**	9.38	15.77	17.52	21.09	21.87	24.11	**0.678**
**Tartarate**	5.44	9.34	10.43	17.47	12.65	24.11	**0.360**
**Malate**	2.05	23.52	10.47	20.61	6.87	13.61	**0.378**
**Succinate**	11.22	20.72	18.61	16.58	13.79	15.30	**0.903**
**Lactate**	110.71	296.19	225.71	263.11	187.80	170.35	**0.316**
**Formate**	2.25	4.70	4.20	3.90	3.87	4.39	**0.977**
**Acetate**	56.56	101.51	35.09	25.46	17.34	16.27	**0.007**

Note. Gangwon; GG, Gyeonggi; CC, Chungcheong; JL, Jeolla; GS, Gyeongsang; JJ, Jeju provinces.

Table S4. Mean value of regional *ganjang* products for sensory attributes and *p*-value derived from analysis of variance.

	GW	GG	CC	JL	GS	JJ	*p*-value
**Color_A**	3.57	5.20	4.15	4.85	3.10	4.48	**0.154**
**Pungent_O**	2.98	2.95	3.00	2.88	3.06	3.07	**0.918**
**Alcohol_O**	2.35	2.01	2.22	2.08	2.33	2.34	**0.764**
**Briny_O**	4.16	4.17	3.88	3.82	3.66	3.97	**0.204**
**Burnt_O**	2.09	2.74	2.31	2.13	1.92	2.19	**0.401**
**Ferermented_O**	3.46	3.40	3.57	3.30	3.53	3.45	**0.806**
**Fermented fish_O**	3.27	3.28	3.29	3.08	3.19	3.21	**0.940**
**Roasted soybean_O**	2.49	2.63	2.60	2.49	2.51	2.43	**0.973**
**Beany_O**	2.20	2.10	2.23	2.06	2.28	2.20	**0.801**
**Sour_O**	2.42	2.24	2.33	2.28	2.45	2.42	**0.828**
**Sweet_O**	2.10	2.04	2.08	2.25	2.07	2.13	**0.685**
**Dusty_O**	1.67	1.99	1.70	1.64	1.65	1.72	**0.511**
**Meju_O**	3.47	3.39	3.56	3.23	3.50	3.41	**0.689**
**Grain_O**	2.19	2.25	2.39	2.22	2.33	2.14	**0.723**
**Chemical_O**	1.74	1.84	1.79	1.63	2.02	2.04	**0.410**
**Sweetness**	2.43	2.31	2.45	2.63	2.51	2.54	**0.564**
**Sourness**	2.55	2.46	2.56	2.60	2.66	2.64	**0.645**
**Saltiness**	4.88	5.10	4.88	4.81	4.76	4.95	**0.444**
**Bitterness**	2.24	2.58	2.28	2.27	2.13	2.27	**0.385**
**Umami**	3.03	2.98	3.07	3.22	3.06	3.16	**0.900**
**Chemical_F**	1.66	1.80	1.69	1.56	1.81	1.90	**0.380**
**Alcohol_F**	1.74	1.66	1.79	1.70	1.82	1.79	**0.906**
**Fermented_F**	2.93	3.01	3.18	3.02	3.05	2.96	**0.580**
**Fermented fish_F**	3.16	3.28	3.25	3.09	3.11	3.20	**0.907**
**Meju_F**	3.11	3.00	3.25	2.97	3.22	3.10	**0.514**
**Roasted soybean_F**	2.23	2.24	2.31	2.23	2.27	2.20	**0.992**
**Beany_F**	1.83	1.82	1.98	1.77	1.92	1.90	**0.712**
**Burnt_F**	1.76	2.39	1.83	1.75	1.60	1.77	**0.306**
**Astringent_M**	2.07	2.43	2.13	1.94	1.92	2.09	**0.042**
**Metallic_M**	1.58	1.72	1.52	1.51	1.53	1.61	**0.398**
**Biting_M**	2.23	2.64	2.36	2.23	2.25	2.23	**0.371**
**Body_M**	2.39	2.97	2.57	2.83	2.39	2.66	**0.240**
**Sweet_AT**	2.30	2.25	2.40	2.59	2.37	2.50	**0.477**
**Sour_AT**	2.15	2.09	2.06	2.10	2.12	2.23	**0.852**
**Salty_AT**	4.22	4.29	4.12	4.01	4.01	4.23	**0.386**
**Bitter_AT**	1.94	2.37	1.97	2.03	1.92	2.07	**0.200**
**Umami_AT**	2.80	2.80	2.78	2.97	2.76	2.89	**0.879**

Note. _A, appearance; _O, odor; _F, flavor; _M, mouthfeel; _AT, aftertaste; GW, Gangwon; GG, Gyeonggi; CC, Chungcheong; JL, Jeolla; GS, Gyeongsang; JJ, Jeju provinces.

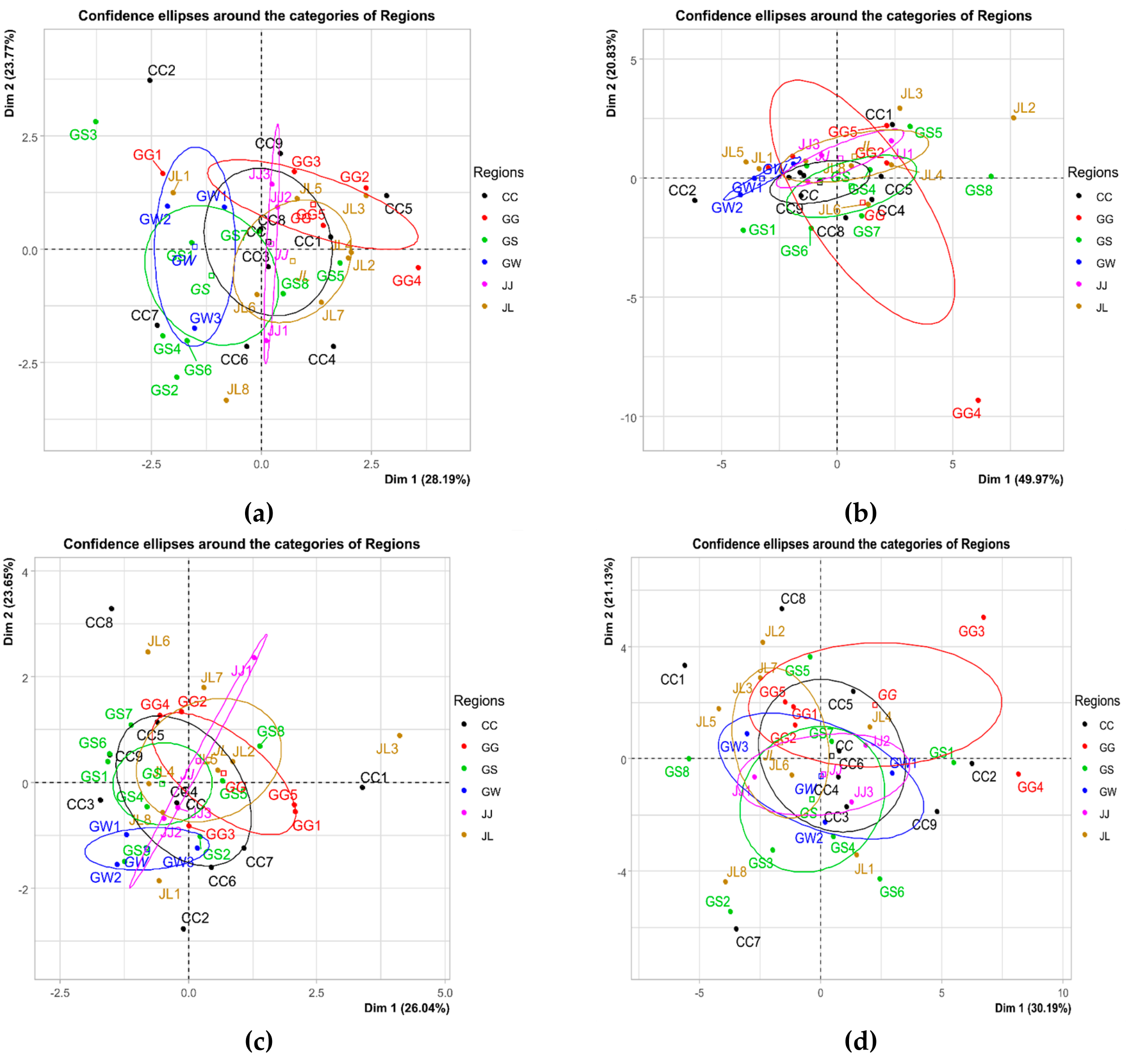

Figure S1. Confidence ellipses of (a) general physicochemical properties; (b) free amino acids; (c) organic acids; and (d) sensory attributes configuration of categories by producing regions producing regions. GW, Gangwon; GG, Gyeonggi; CC, Chungcheong; JL, Jeolla; GS, Gyeongsang; JJ, Jeju provinces.
